# East Texas Medical Association

**Published:** 1888-03

**Authors:** J. E. Mayfield

**Affiliations:** Secretary E. T. Med. Association


### SOCIETY NOTES.

### EAST TEXAS MEDICAL ASSOCIATION.

*To the Medical Profession of East Texas and Adjacent Territory :*

On the 17th day of November, 1886, the East Texas Medical Association was organized in the town of Nocogdoches, in response to a call by the profession of this place. Its progress has been auspicious until recently. It now has sixty names on the roll of members. But from causes and in ways that I need not now recite, it has got out of its legitimate channel, so far as to amount almost to disorganization.

Consultation with numerous members has resulted in the decision that we meet and reorganize in the town of Nacogdoches, on Tuesday night, April 3, 1888. To reorganize, to adopt new laws, make a new roll of members, and in short, to rub out and begin over.

I earnestly invite all interested to attend, and to use every means to promote the cause. Original papers are especially solicited; those heretofore solicited are still called for.

The profession at Nacogdoches heartily join in this invitation, Come and you will be glad of it.

J. E. MAYFIELD,
Secretary E. T. Med. Association.

